# Impact of straw return on greenhouse gas emissions from maize fields in China: meta-analysis

**DOI:** 10.3389/fpls.2025.1493357

**Published:** 2025-02-26

**Authors:** Qi Sun, Xue-jia Gu, Yu-feng Wang, Hong-sheng Gao, Xiao-jun Wang, Xue-li Chen, Si-miao Sun

**Affiliations:** ^1^ Heilongjiang Academy of Black Soil Conservation & Utilization, Harbin, China; ^2^ Key Laboratory of Agro-Environment in Northeast Plain, Ministry of Agriculture and Rural Affairs, Harbin, China

**Keywords:** straw return, greenhouse gases, maize, meta-analysis, random forest

## Abstract

**Introduction:**

An increase in the amount of greenhouse gases (GHGs) in the atmosphere causes global warming, and >14% of all GHG emissions come from agricultural activities. The three primary atmospheric GHGs are CO_2_, CH_4_, and N_2_O; therefore, regulating GHG emissions from agroecosystems is important for global climate management. Straw return is an environmentally friendly agricultural practice that positively affects crop production and soil fertility. However, its effects on long-term GHG emissions remain controversial.

**Methods:**

To examine the impact of straw return on GHG emissions from Chinese maize fields, 281 data pairs from 45 publications were assessed using a data meta-analysis.

**Results:**

The findings demonstrated substantial increases in CO_2_ and N_2_O emissions of 140 and 40%, respectively. Methane emissions increased by 3% after straw return, and the maximum effect value of CO_2_ emissions was 2.66 at nitrogen rates<150 kg/hm^2^. The effect value of CH_4_ emissions increased with an decrease in soil organic content, and the effect value of CH_4_ emissions changed from negative to positive at concentrations >6 g/kg. With a nitrogen rate increase, N_2_O emission effects under straw return initially increased and then decreased. N_2_O emissions increased significantly when nitrogen rates were<250 kg/hm^2^. The results of a random forest model showed that the most important factor affecting CO_2_ and N_2_O emissions from corn fields under straw return was the amount of nitrogen applied, and the most important factor affecting CH_4_ emissions from corn fields under straw return was soil organic carbon content.

**Discussion:**

This shows that a suitable straw return can achieve the mutually beneficial goal of guaranteeing food security and minimizing adverse effects on the environment.

## 1 Introduction

Global population is projected to increase by 20% over the next 20 years, posing a serious threat to environmental sustainability and food security (Huang et al., 2021). Increased amounts of food have been produced in agriculture to fulfill the expanding demand, resulting in large amounts of crop residue (Ali et al., 2019), with straw being the primary source. Currently, agriculture in China produces nearly 800 million tons of straw, and this number is increasing (Sun et al., 2019). Straw treatment methods include returning to the field, preparing biochar, making fuel and animal feed, and field burning (Bhattacharyya et al., 2021).

Returning straw to the field is an economical, efficient, and environmentally friendly way of straw treatment (Jin et al., 2020), providing crops with essential nutrients such as N, P, K, and various micronutrients necessary for growth (Mabagala et al., 2020). Returning straw to the field can improve soil nitrogen cycling and decrease soil erosion—among other ecosystem services (Yin et al., 2018)—and regulate the C-N balance in the soil and alter microbial activity, thereby affecting greenhouse gas (GHG) emissions. Maize is currently one of the most important global grain crops and the first of the three major grain crops in China. In 2020, the total production of corn was 260.67 million tons, accounting for 38.94% of the total annual grain production and 42.27% of the total production of the three major grain crops such as rice, wheat, and corn (Jin-gang et al., 2021). Maize has a sown area of ~20% of the total cultivated area (Meng et al., 2013; Qi et al., 2018) and is mainly grown in the Chinese regions of Heilongjiang, Jilin, Liaoning, Inner Mongolia, Ningxia, Henan, Shandong, Gansu, Shaanxi, and Shanxi (He and Zhou, 2012). Maize absorbs atmospheric CO_2_ through photosynthesis, while CO_2_ is emitted through soil and plant respiration (Gołasa et al., 2021).

Agricultural activities are major contributors to GHG emissions, accounting for >20–25% of global emissions (Tubiello et al., 2021). Thus, enhancing agricultural management techniques is essential for improving the Earth’s climate. Returning straw to the field modifies the physical, chemical, and biological characteristics of the soil, which in turn affects soil GHG emissions (Wang et al., 2019; Hou et al., 2020). CO_2_ is one of the most important GHGs contributing to anthropogenic climate change (Gómez Gallego, 2022). Arable soils emit CO_2_ through the decomposition of crop residues, crop root respiration, and the mineralization of soil organic carbon (SOC) (Liu et al., 2014). Methane-oxidizing bacteria in the soil can oxidize CH_4_ under aerobic conditions, making drylands a sink for atmospheric CH_4_ (Lafuente et al., 2019). These bacteria are a class of gram-negative bacteria that oxidize CH_4_ through the action of CH_4_ monooxygenase and dehydrogenase, using CH_4_ as their sole source of carbon and energy (Jhala et al., 2014). The oxidation of CH_4_ and NH_3_ to NO_2_^-^ is catalyzed by CH_4_ monooxygenase, and when the soil NH_3_ content is high, CH_4_ emissions is promoted (Lee et al., 2009). Past studies have shown that methane and nitrous oxide emissions increase significantly in more permeable soils and at higher temperatures, so that there is some variation in GHG emissions across soil types and average annual temperatures (Fan et al., 2021; Yan et al., 2022). Methane emissions rise with increasing soil moisture, with nitrous oxide emissions reaching a maximum when soil moisture content is about one quarter (Laville et al., 2011). Soil temperature and moisture are determined by climatic conditions. Similarly, the amount of nitrogen rates to the soil has a significant effect on nitrous oxide emissions (Fan et al., 2022). In addition, tillage and soil pH also affect GHG emissions. Overall, the process of GHG emissions is a complex interaction of multiple factors therefore, a comprehensive analysis of the effects of straw return on GHG emissions from maize under different conditions is needed. This study found that returning straw to the field decreased CH_4_ uptake and increased CH_4_ emissions, in line with the findings of (Hu et al., 2016). This may be because straw provides a carbon source for methanogenic bacteria, enhancing their activity. Straw decomposition consumes a large amount of oxygen, creating an anaerobic environment that favors the decomposition of organic matter by methanogenic bacteria that release CH_4_. Studies have shown that no-tillage practices decrease CH_4_ emissions, whereas tillage treatments reduce it. This is because no-tillage increases soil porosity, which enhances the gas diffusion rate and promotes CH_4_ oxidation (Zhang et al., 2013).

Straw serves as a crucial vector of substances, energy, and nutrients, endowing it with considerable value as a natural resource. The practice of directly returning straw to the field is currently the primary method of straw utilization (Zuliang et al., 2019), and also one of the principal agricultural strategies to enhance soil fertility and increase crop yield (Chen et al., 2020). However, this also leads to problems such as poor sowing quality, competition for resources with fodder, widespread pests, diseases, and grasses, and impacts on GHG emissions (Ting et al., 2017). While previous studies have indicated that returning straw to the field can stimulate the emission of CO_2_ and CH_4_ (Wu et al., 2022), the impact on N_2_O emissions remains ambiguous and is closely related to factors such as soil characteristics, the quantity of straw returned to the field, the method of straw incorporation, and post-straw application water and fertilizer management (Chan et al., 2002). For instance, returning straw to the field mitigates N_2_O emissions from wheat fields in the later season, whereas under continuous flooding methods, straw application does not lower N_2_O emissions (Jianwen et al., 2003). Conversely, returning straw to the field might exacerbate the emission of soil N_2_O (Guoyuan et al., 2001).

Emissions of N_2_O from agricultural soils primarily occur through chemical, biological, and denitrification processes. Additional factors that increase N_2_O emissions include the use of straw, manure, and synthetic nitrogen fertilizers (Akhtar et al., 2020). Currently, most research focuses on how straw return affects GHG emissions from paddy fields. However, very few studies have examined how straw return affects GHG emissions from maize fields. Therefore, there is an urgent need to improve soil fertility and reduce GHG emissions. This study used a meta-analysis to comprehensively examine the effects of straw return conditions on GHG emissions. The aim was to provide a reference for GHG emission reduction in maize planting technology innovation.

## 2 Data analyses and methods

### 2.1 Data selection

**Figure 1:**
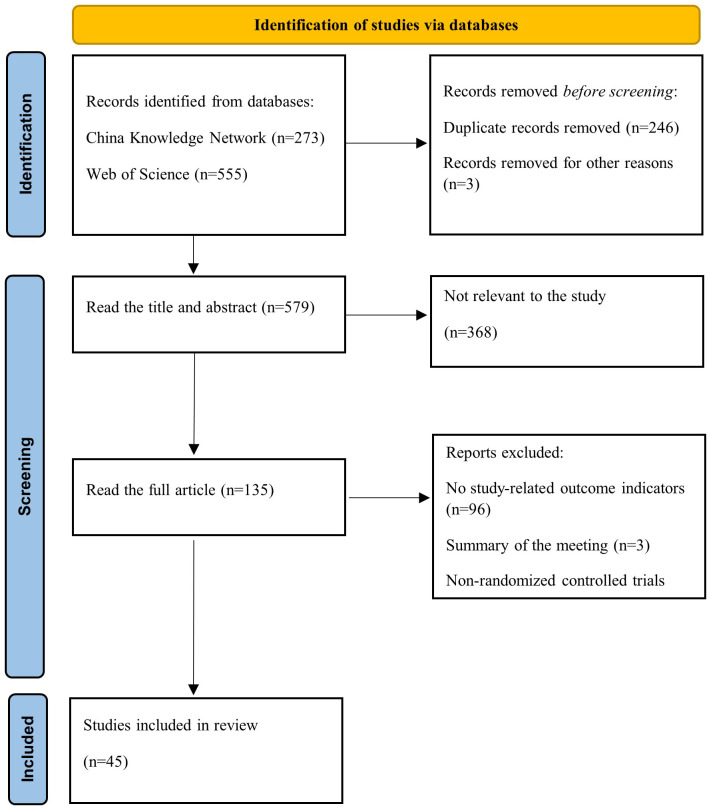
Searching and filtering flowcharts.

**Figure 2:**
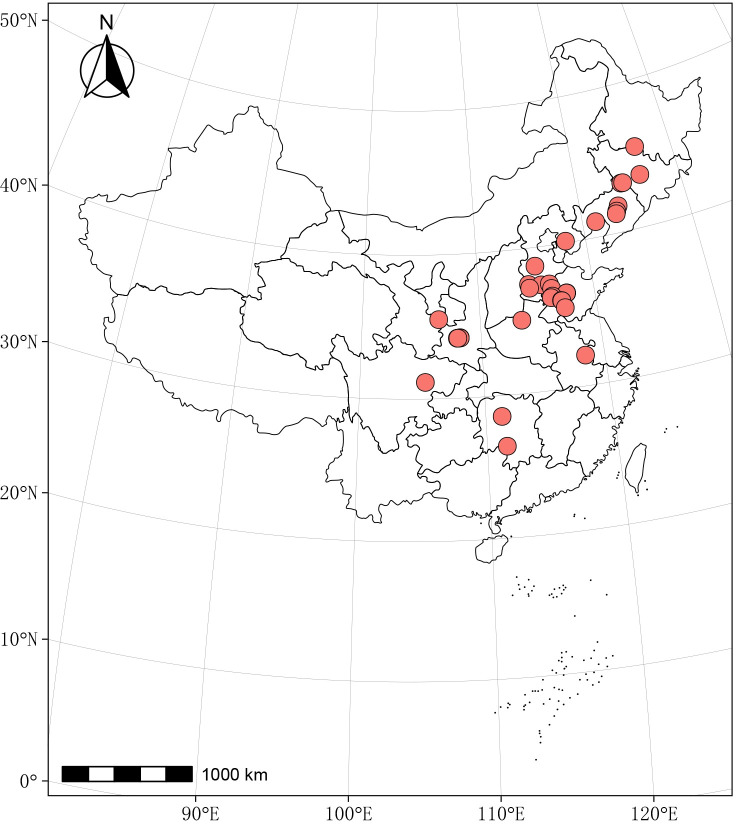
The geographical distribution of the trial locations in the meta-analysis.

This study searched the China Knowledge Network (CNN) and Web of Science for articles on GHG emissions from cornfield experiments using straw mulching. Literature containing the terms “straw return,” “straw mulching,” “greenhouse gas,” and “maize” in the title, keywords, or abstract prior to December 2023 was gathered (**Figure 1**). The following study criteria for a meta-analysis were identified: (i) the study was an in-field experiment within China, with maize as the planting crop and no less than three replications; (ii) cumulative emissions and standard deviations of one or more of CO_2_, CH_4_, and N_2_O, as well as the location of the experiment, nitrogen rate, plant method, tillage method, average annual air temperature, rainfall, and basic soil conditions were reported (**Figure 2**); (iii) the research was conducted in the same field with the same crop and soil conditions. If there were two growing seasons, each growing season was included as a separate observation period. Forty-five articles that met the inclusion criteria were screened and 281 data pairs were extracted. Of the total dataset, 40 % did not show variance in the mean. For these datasets, one-tenth of the mean was used to perform the meta-analysis. The values for each variable were obtained directly from tables, text, or graphs using the GetData Graph Digitizer V.2.22. When conducting a meta-analysis, it is important to ensure that the individual observations are statistically independent (Li et al., 2017).

### 2.2 Data analysis

Summary of the overall effects of straw return in maize fields on CO_2_, CH_4_, and N_2_O emissions, we refer to Gui and He et al (Gui et al., 2024; He et al., 2024). In the dataset here, there were eight predictor variables: total nitrogen (g/kg), soil organic carbon (g/kg), nitrogen rate (kg/hm^2^), soil pH, soil type, average annual temperature (°C), rainfall (mm), plant method, and tillage method. The potential of these variables to emit GHGs under straw return conditions was assessed using response ratios (R) (Hedges, 1999). Soil types were classified according to categories found in the literature: cinnamon, brown, saline-alkali, fluvo-aquic, black, dark loessial, red soil, yellow-brown, and purple. The nitrogen rate was categorized into four levels:<150, 150–250, 250–350, and >350 kg/hm^2^. The analysis included data on soil TN (total nitrogen) content, categorized into three levels:<1, 1–1.5, and >1.5 g/kg. SOC (soil organic carbon) was also analyzed and categorized into three levels:<6, 6–12, and >12 g/kg. Farming practices were classified as tillage or no-tillage. In addition, two planting methods were compared: continuous and rotational. Rainfall was classified into three levels of<400, 400–800, and >800 mm; the effects at three different annual average temperatures of<10, 10–15, and >15°C were compared; and research on how straw return affects GHG emissions was conducted at soil pH of<6, 6–8, and >8.

In instances where the research report does not provide the standard deviation (S) or the standard error (Se), a value of one-tenth of the mean is used as a surrogate. When the dataset includes both the standard error (Se) and the number of replications (n), the standard deviation (S) is derived using the following formula:


(1) $$S=\sqrt{n}\times S_{e}$$

The standard mean difference (SMD) was used to quantify the effects of GHG emission under straw return, which were calculated using the following equation:


(2) $$SMD=\frac{\bar{X}\text{n}1-\bar{X}\text{n}2}{\text{SC}}(1-\frac{3}{4(a1+a2-2)})$$

(3) $$SC=\sqrt{\frac{Sd1^{2}(a1-1)+Sd2^{2}(a2-1)}{\left(a1+a2-2\right)}}$$

Here, a1 and a2 denote the number of replicates for the experimental and control groups, respectively. Sd_1_ and Sd_2_ are the standard deviations of GHG emissions for the experimental and control groups, respectively.X_n1_ and X_n2_ represent the mean GHG emissions for the experimental and control groups, respectively. The variance (var) of X_n_ is determined as follows:


(4) $$V\text{ar}=\frac{a1+a2}{a1\times a2}(\frac{SMD^{2}}{2(a1+a2)})$$

A meta-analysis was carried out using the “Metafor” package version 4.6-0 in the R environment (v4.4.0; http://www.r-project.org/) for data processing and analysis using the “forestplot” package version 1.2-5 for forest plotting. The mean effect value of straw return on GHG emissions was estimated using a random-effects model. The SMD method was applied to calculate the mean GHG emission effect value, p-value, and 95% confidence interval (95% CI). The mean differences were standardized using published variance and repeated data. Permutation tests were conducted to validate the robustness of the results. Hedges’ adjustment (g) was used for the SMD (Cooper, 1994). Heterogeneity was assessed by estimating τ^2^ using the DerSimonea–Laird estimator and applying a Knapp–Haddon adjustment. Confidence intervals (CI) were used for τ^2^. The input data included the mean value of GHG emissions from soil with and without straw, along with the corresponding standard deviation and number of samples. When the 95% CI contained 0, there was no significant effect on GHG emissions (*P* > 0.05). A 95% CI >0 suggests a substantial impact on GHG emissions (*P*< 0.05). If the 95% CI was<0, it implied a significant inhibition of GHG emissions (*P*< 0.05).

This research employed a random forest model in which three GHG emissions were used as dependent variables, while environmental factors such as NR, pH, TN, soil, SOC, average annual temperature (AAT), plant method, tillage method, and rainfall were incorporated as independent variables. The influence of these environmental elements on GHG emissions was assessed by considering the importance scores of the input parameters and the significance of their effects. The random forest model was implemented using the randomForest package version 4.7-1.2 in the R environment (v4.4.0; http://www.r-project.org/).

### 2.3 Publication bias

We plot funnel plot graphs to test for publication bias. Here, a funnel plot is a simple scatterplot showing the relationship between the effect size of an individual study and some measure of its precision or sample size for each study. The shape of the scatterplot should resemble a symmetrical inverted funnel with a wide base and a narrow top. Trim and fill methods were used to adjust the final results of the meta-analysis. The distribution of all studied effect measures in this paper is symmetric (**Supplementary Figures S1–S3**).

## 3 Results and analyses

### 3.1 Changes in GHGs under straw return conditions

**Figure 3:**
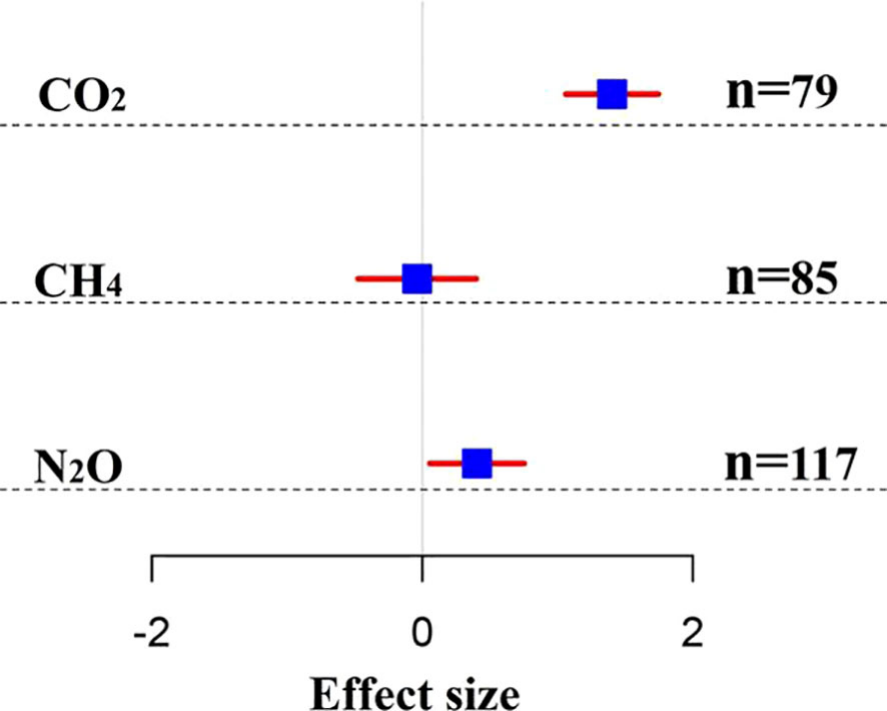
Summary of the overall effects of straw return in maize fields on CO_2_, CH_4_, and N_2_O emissions. Blue squares are effect values; red line segments are 95% confidence intervals.

Returning straw to the field resulted in a considerable increase in soil CO_2_ emissions, with a mean effect size of 1.40 (a 140% increase in CO_2_ emissions) after returning straw to the field compared with the control group (*P*< 0.05). Returning straw to the field increase CH_4_ emissions by an average of 3% (95% CI: -0.47 to 0.39) compared to not returning it to the field (**Figure 3**; **Supplementary Table S1**). Returning straw to the field increased N_2_O emissions by 40% compared with not returning straw.

### 3.2 Changes in CO_2_ emissions under straw return conditions

**Figure 4:**
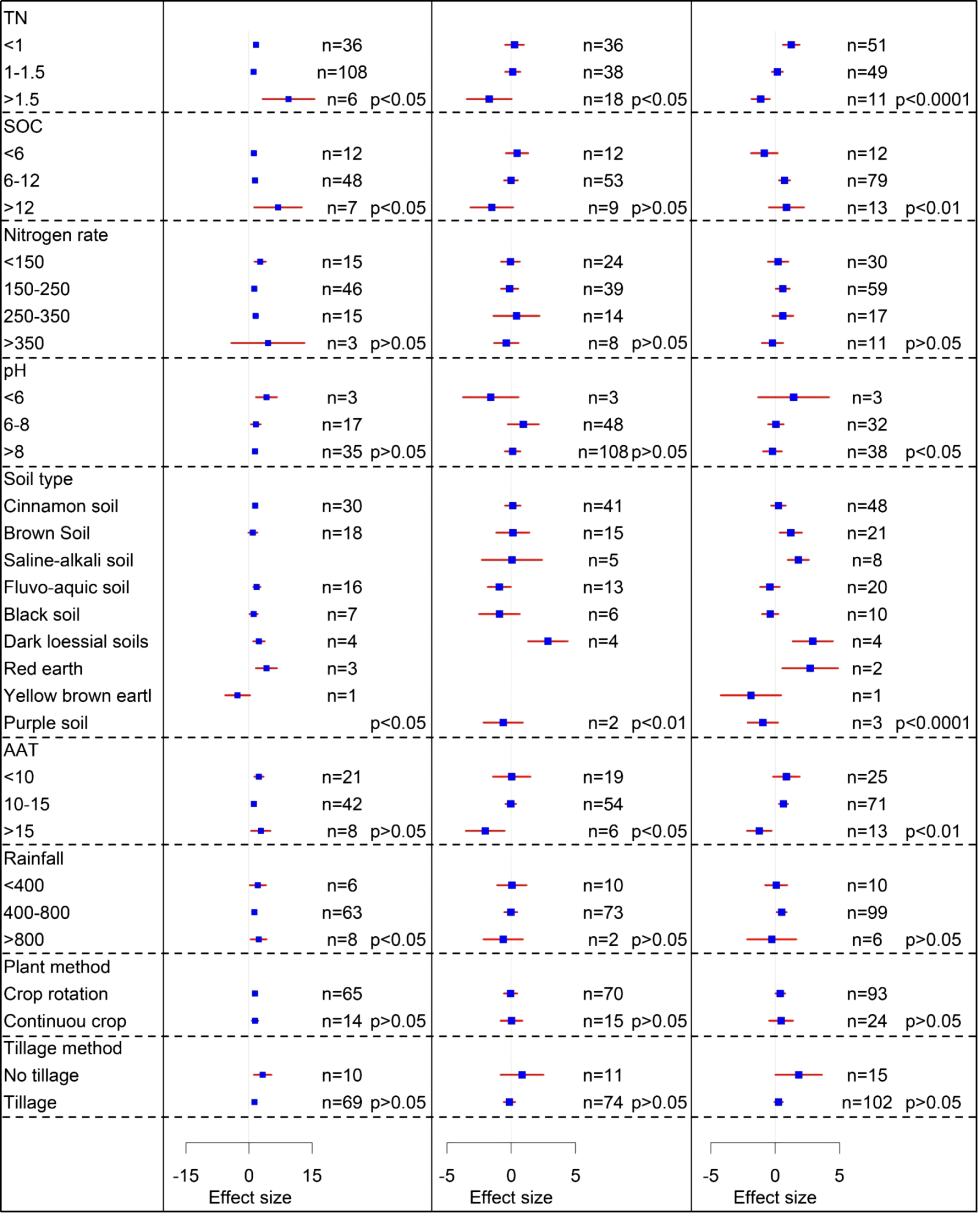
The effects of TN (g/kg), SOC (g/kg), nitrogen rate (kg/hm^2^), soil pH, soil type, average annual temperature (°C), rainfall (mm), plant method, and tillage method on CO_2_, CH_4_, and N_2_O emissions from straw return in maize fields. Blue squares are effect values; red line segments are 95% confidence intervals.

As shown in **Figure 4**; **Supplementary Table S2**, red soil had the greatest positive impact of straw return on CO_2_ emissions among the various soil types (4.14), there was a negative impact for yellow-brown soil (-2.72), and straw return on cinnamon soil (1.46), fluvo-aquic soil (1.85), black soil (1.1), and dark loessial soil (2.38) all contributed to CO_2_ emissions. Under the condition of straw return to the field, the CO_2_ emission effect value of continuous cropping (1.45) is higher than that of crop rotation (1.38). Tillage significantly increased CO_2_ emissions from the soil after straw return (1.31, *P*< 0.05), whereas no tillage had a more significant average increase of 325% in CO_2_ emissions (*P*< 0.05). A nitrogen rate of 150–250 kg/hm^2^ significantly increased CO_2_ emissions with an effect size of 1.24 (*P*< 0.05). Straw return to the field significantly contributed to CO_2_ emissions from soils with different pH values (*P*< 0.05), with an average effect size of 2.4. As the soil pH increased, the impact of CO_2_ emissions decreased with the largest average effect value of 4.14 and a soil pH<6. Effect values were 1.63 and 1.45 at a soil pH 6–8 and >8. The effect of straw on CO_2_ emissions was significantly affected by different average annual temperatures, with an average effect value of 2.10 (*P*< 0.05). The average effect value was the largest at an average annual temperature of >15°C, with an average effect value of 2.82. The effect of straw on CO_2_ emissions was significant at all rainfall levels (*P*< 0.05). The average effect was 2.3 for annual rainfall >800 mm, and the average effect was 2.12 and 1.25 when the rainfall was<400 mm and 400–800 mm. As the SOC increased, the effect value of CO_2_ emissions gradually increased, and the effect of straw returning to the field on CO_2_ emissions was significant compared with that of straw not returned to the field (*P*< 0.05). The effect values were 4.14, 1.63, and 1.41 at SOC of<6, 6–8, and >8, respectively (**Figure 4**; **Supplementary Table S2**).

### 3.3 Changes in CH_4_ emissions under straw return conditions

Straw return significantly reduced the CH_4_ emissions in fluvo-aquic soils by an average of 92% across soil types (*P*< 0.05) (**Figure 4**; **Supplementary Table S2**). Continuous cropping after straw return (-0.02) had a lower effect value than crop rotation (0.04), making it more effective in decreasing CH_4_ emissions. Among the different nitrogen rates, the largest effect value (0.38) was found at nitrogen rates >350 kg/hm^2^, and the smallest effect value (-0.4) was found at nitrogen rates between 250 and 350 kg/hm^2^. When the soil pH was >6 and<6, the return of straw to the field decreased and increased the emissions of CH_4_, respectively. The effect of straw return to the field on soil CH_4_ emissions was greatest when the pH was between 6 and 8 (-0.94). Regarding the mean annual air temperature, the return of straw to the field had a significant effect on the CH_4_ emissions (*P*< 0.05). The effect of straw return on the CH_4_ emissions decreased as the mean annual air temperature increased. At temperatures >15°C, straw return significantly increase CH_4_ emissions (-2.02, *P*< 0.05). In all the collected organic carbon data, it was found that elevated SOC after straw return significantly promoted CH_4_ emissions (*P*< 0.05). Additionally, straw return promoted CH_4_ emissions when SOC content was >12 g/kg (1.51) (**Figure 4**; **Supplementary Table S3**).

### 3.4 Changes in N_2_O emissions under straw return conditions

The contribution of straw to N_2_O emissions varied among soil types (**Figure 4**; **Supplementary Table S4**). Straw significantly increased N_2_O emissions from brown soil, saline-alkali soil, dark loess soil, and red soil by 1.21, 1.8, 2.93, and 2.72, respectively (*P*< 0.05). N_2_O emissions also increased significantly by 40% under crop rotation conditions (*P*< 0.05). No tillage led to a significant increase in N_2_O emissions after straw return (1.83, *P*< 0.05). With the increase in nitrogen rates, the effect value of N_2_O emissions showed an increasing and then a decreasing trend. Straw return to the field at nitrogen rates of 150–250 kg/hm^2^ significantly increased N_2_O emissions, with an average increase of 60% (*P*< 0.05). The effect of straw return on N_2_O emissions varied with annual average temperatures, and the effect decreased as the temperature increased. N_2_O emissions increased by 65% at temperatures of 10–15°C and decreased at temperatures >15°C (-1.23). Rainfall of 400–800 mm also increased N_2_O emissions (0.51). Increasing the SOC content had a larger effect on N_2_O emissions, and straw return to the field increased N_2_O emissions by 73% at a SOC content of 6–12 g/kg. The impact of soil total nitrogen content on N_2_O emissions decreased. Specifically, N_2_O emission increased by 125% at<1 g/kg and decreased significantly at >1.5 g/kg (-1.12, *P*< 0.05) (**Figure 4**; **Supplementary Table S4**).

### 3.5 Influence factors of straw returning to the field on soil GHG emissions

**Figure 5:**
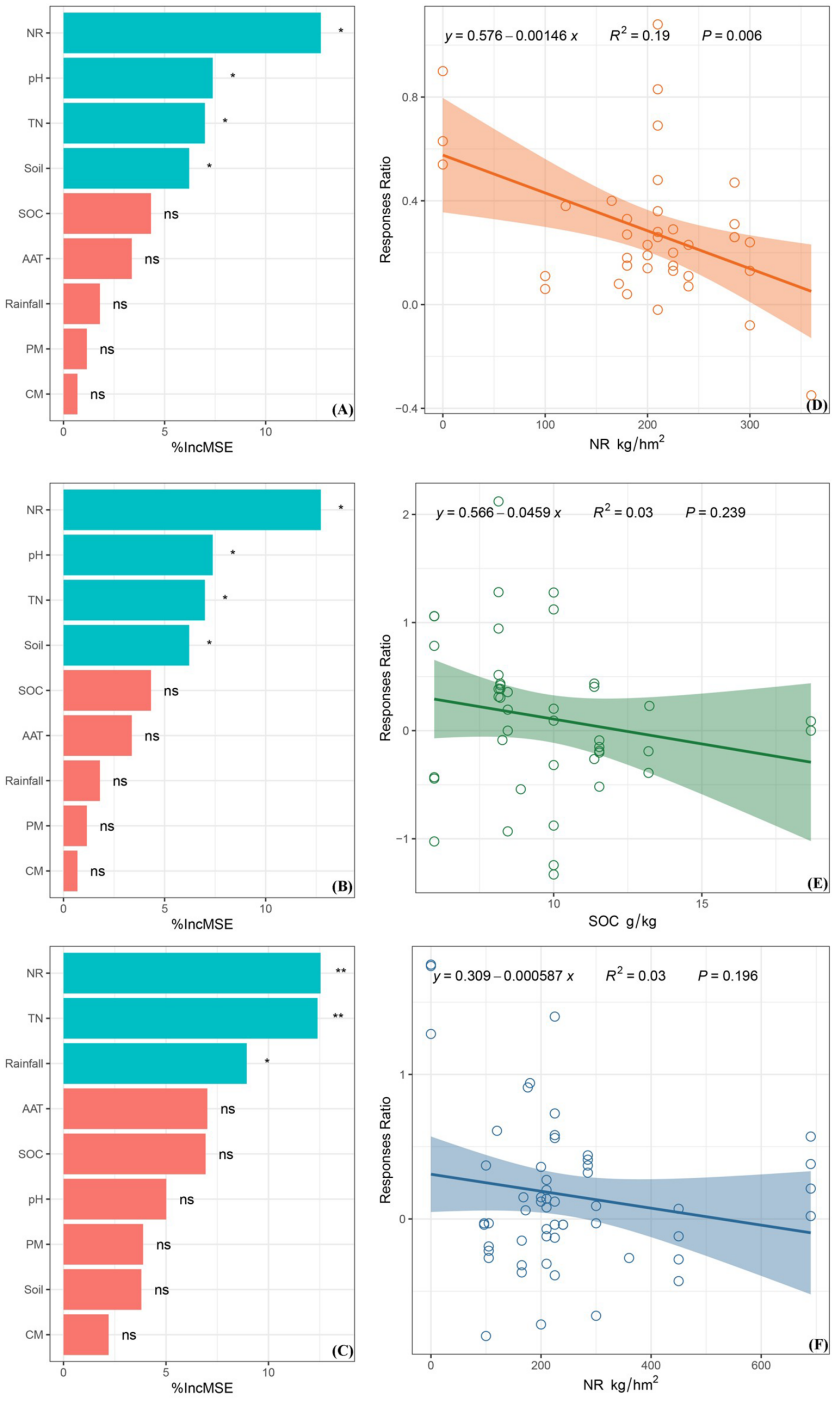
The figure on the left illustrates the factors that impact greenhouse gas (GHG) emissions from soils where straw was returned. On the right side, the figure displays the relationship between CO_2_ emissions and nitrogen fertilization rate **(A, D)**, CH_4_ emissions and organic carbon content **(B, E)**, and N_2_O emissions and nitrogen fertilization rate **(C, F)** under straw-returned conditions. "ns" indicates no significant difference, "*" indicates a significant difference (P < 0.05), and "**" indicates a strong significant difference (P < 0.01).

The random forest model predicts variables, and its average importance indicated that nitrogen rate, soil pH, soil total nitrogen content, and soil type were significant predictors of CO_2_ emissions from maize land affected by straw return. SOC content was found to be a significant predictor of CH_4_ emissions in maize fields affected by straw return. The soil nitrogen rate, total nitrogen content, and rainfall were important factors affecting the magnitude of the effect of N_2_O emissions from maize land after straw return (**Figure 5**). After straw return, the nitrogen rate had the greatest effect on CO_2_ and N_2_O emissions. Finally, SOC content was identified as the primary factor affecting CH_4_ emissions in relation to straw return.

This study found that after the straw was returned to the field, there was a negative correlation between the CO_2_ emission flux and nitrogen rate, and a substantial association between the change in nitrogen rate and the strength of the CO_2_ emission effect (*P*< 0.05, *R^2^* = 0.19). These results suggest that the CO_2_ emission effect tends to decrease under different nitrogen rates. The fitted curve and response ratio line did not intersect, indicating that the effect size of CO_2_ emissions decreased with increasing nitrogen rates under straw return compared to straw non-return (**Figures 5A, D**). CH_4_ fluxes varied significantly under different soil total nitrogen content conditions, and the CH_4_ effect value decreased as the SOC content increased. The intersection point of the fitted curve and the response ratio line was zero when the soil total nitrogen content was 12.33 g/kg. This indicates that CH_4_ emissions under straw return was not affected by SOC content compared to straw that was not returned to the field (**Figures 5B, E**). The effect of N_2_O was negative, with a downward-sloping curve fitted to the nitrogen rate. At nitrogen rates of 526.41 kg/hm^2^, the effect value was 0, indicating that N_2_O emissions from maize fields was not affected by nitrogen rates under straw return. When compared to straw that was not returned to the field, the effect value of N_2_O emissions decreased with increasing nitrogen rates (**Figures 5C, F**).

## 4 Discussion

**Figure 6:**
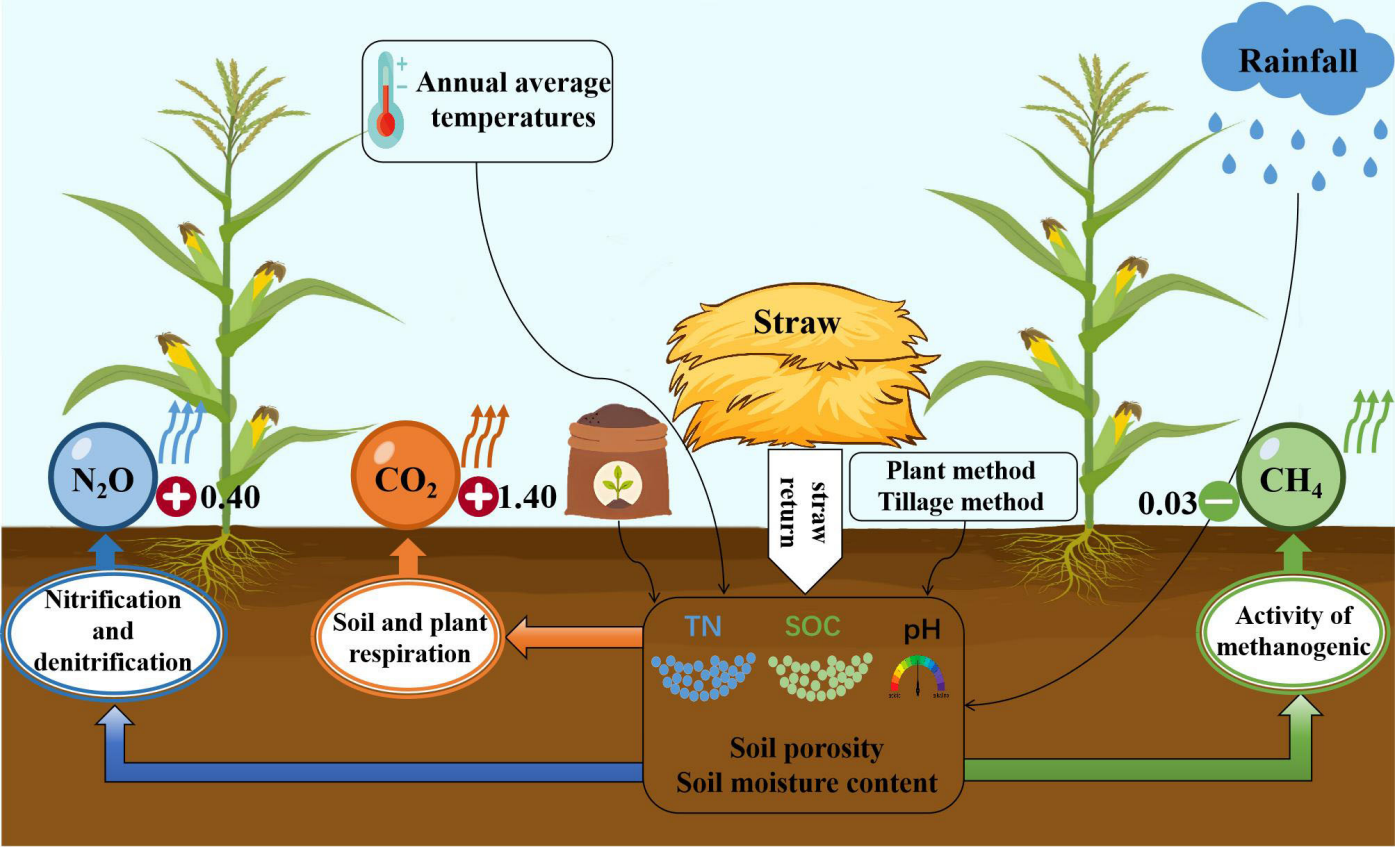
Conceptual map of the impact of straw returning on greenhouse gas emissions. The plus sign in red indicates an increase, while the minus sign in green indicates a decrease. The number next to the sign represents the corresponding effect value.

Two main parts are discussed here. First, the effects of straw return on the three types of GHG emissions and the results of the meta-regression were specifically analyzed, considering the differences in environmental factors and soil conditions. Second, the shortcomings of this meta-regression analysis are summarized and suggestions are made to improve the study in the future. Subgroup and meta-regression analyses were carried out, and variability results were obtained, providing a theoretical foundation for future justifications for straw returns (**Figure 6**).

### 4.1 The effects of straw return on CO_2_ emissions

This study demonstrated that CO_2_ emissions increased significantly, with an average increase of 140%, after straw was returned to the field. This was likely due to the significant increase in crop productivity, which resulted in increased root respiration (Ben-Noah and Friedman, 2018). Additionally, changes in the microbial biomass resulting from straw return may contribute to increased carbon emissions. Under straw return conditions, tillage treatments accelerate organic matter decomposition and significantly increase CO_2_ emissions (Liying et al., 2018). Enhanced microbial biomass carbon in the soil can stimulate the decomposition of SOC and straw, leading to increased soil CO_2_ emissions (Xie et al., 2021). Straw return increases soil moisture, thereby promoting CO_2_ emissions (Wang et al., 2019) and increasing soil porosity (Li et al., 2022), thereby, improving soil diffusivity and increasing soil surface CO_2_ emissions (Fan et al., 2020). It is essential to remember that these findings were based on objective evaluations and subject to specific soil characteristics and conditions. Additionally, it may enhance the carry-over function of sandy soils and increase soil water content because of soil agglomeration and the high water absorption capacity of organic matter (Skaalsveen et al., 2019). However, in dense clay soils, the return of straw to the soil enhances its organic carbon content, which increases soil porosity and CO_2_ emissions (Wang et al., 2021a).

Straw return can regulate soil CO_2_ emissions by affecting soil temperature, water content, and microbial population and activity. Higher temperatures caused by the return of straw have the potential to accelerate the breakdown of organic materials, boost microbial development and activity, and increase CO_2_ emissions (Wu et al., 2022). The results of the model importance analysis indicated that the amount of nitrogen applied had the greatest impact on the magnitude of CO_2_ emissions under straw return. This finding is consistent with those of Huiyi et al., who demonstrated that the soil respiration rate was significantly affected by the amount of applied nitrogen and increased with the amount of nitrogen applied (Huiyi et al., 2009; Peiyu et al., 2011).

### 4.2 The effect of straw return on CH_4_ emissions

Tillage treatment can lead to soil compactness, reduced soil infiltration rate, weakened soil gas diffusion capacity, and anaerobic environments, ultimately resulting in an increase in cumulative CH_4_ emissions. It is noteworthy that the impact of CH_4_ emissions under straw return is sensitive to changes in soil pH. Straw return has the potential to increase soil pH, transforming it to a neutral or slightly alkaline state and increasing the activity of methanogenic bacteria, which in turn promotes CH_4_ emissions. Adding nitrogen to the soil can improve crop growth and photosynthesis, promote the secretion of photosynthetic products, and provide metabolic substrates for methanogenic bacteria, thereby enhancing microbial activity. SOC is a crucial indicator of soil nutrient content, and the model importance analysis showed that SOC content was the most notable factor affecting CH_4_ emissions in cornfields under straw return conditions. Numerous studies have shown that methanogenic bacteria rely exclusively on SOC as metabolic substrates and energy sources. CH_4_ emissions are correlated with SOC to a certain extent (Chu et al., 2015), and returning straw to the field has both positive and negative environmental effects. On the one hand, it could accelerate the decomposition of soil organic matter by increasing microbial adaptation to the environment (Wang et al., 2021b); on the other, the organic carbon present in straw may promoted the emissions of CH_4_. Therefore, it is crucial to consider both of these factors when determining whether to reintroduce straw into fields.

### 4.3 The effects of straw return on N_2_O emissions

N_2_O is produced by microorganisms involved in both nitrifying and denitrifying soil processes (Yang et al., 2022). Soil conditions such as temperature, water content, pH, organic matter, and management practices (fertilizer application, irrigation, and tillage) primarily drive these processes. Straw return can efficiently provide carbon for nitrifying and denitrifying bacteria, affecting the soil environment, carbon and nitrogen content, and soil nitrogen cycling rates. Thus, nitrification and denitrification processes are affected, which eventually influence soil N_2_O emissions (Wang et al., 2021c). The meta-analysis found that straw return significantly increases N_2_O emissions by almost 40%, consistent with the findings of (Wang et al., 2021d). Additionally, consistent with the results of the model significance analysis, the most important factor determining N_2_O emissions under straw return conditions was the amount of nitrogen applied. Nitrification converts nitrogen fertilizers into N_2_O and typically promotes denitrification. Moreover, the interaction between straw return and nitrogen rate can notably affect N_2_O emissions (Xu et al., 2019), and there is a significant positive correlation between soil nitrogen and N_2_O emissions, particularly nitrate nitrogen, under straw return conditions.

Here, it was found that N_2_O emissions increased with an increase in nitrogen rates, but the effect size of N_2_O emissions under the straw return conditions showed a tendency to increase and then decrease with the nitrogen rates. This indicated that the effect of straw return on N_2_O emissions decreased with an increase in nitrogen rates. Under low-nitrogen conditions, straw return can increase the effectiveness of carbon and nitrogen in the soil and improve microbial activity (Xia et al., 2018). However, high-nitrogen conditions are often accompanied by large amounts of N_2_O emissions. At this point, the microbial carbon and nitrogen conditions have been met, and soil carbon and nitrogen are no longer the limiting factors for N_2_O emissions. Straw return to fields can reduce water evaporation and increase soil porosity to improve the water-holding capacity of the soil. A large amount of water can promote the decomposition of straw, owing to an increase in the ratio of soil carbon and nitrogen, and an increase in the soil carbon-to-nitrogen ratio weakens nitrification and denitrification. Soil water content and aeration affect the production and transportation of N_2_O, whereas rainfall markedly affects the soil water and nitrogen conversion processes, ultimately affecting N_2_O emissions (Wei et al., 2022).

## 5 Conclusion

This study analyzed the effect of straw return on GHG emissions from cornfields using a database of published literature. The meta-analysis results indicated a complex interrelationship between straw return and GHG emissions, influenced by region, gas type, nitrogen rate, environmental factors, and soil conditions. Returning straw to the field resulted in a significant increase of 140% in CO_2_ emissions, with nitrogen rate being the main factor affecting this increase. Straw return increased CH_4_ emissions by 3%, with SOC content being the most notable factor affecting CH_4_ emissions. The amount of nitrogen applied was the most important factor affecting N_2_O emissions under straw return conditions. Returning straw to fields increased N_2_O emissions by 40% compared with not returning it. Although much research has been conducted on field straw return, many problems remain, such as subsequent ecological impacts and economic returns. The drawbacks of single-site research can be overcome using meta-analysis, which enables a thorough examination within an area. Nevertheless, there are discrepancies in the research data and experimental designs found in the literature that this study retrieved. Some studies had missing GHG data, rendering it impossible to assess the overall change in the greenhouse effect caused by straw return. Future studies should aim to identify better datasets or utilize process-based models such as denitrification decomposition models to accurately forecast crop growth, yield, and GHG emissions under straw return conditions.

## Data Availability

The original contributions presented in the study are included in the article/**Supplementary Material**. Further inquiries can be directed to the corresponding author.
