# Supplementary material for: Impact of straw return on greenhouse gas emissions from maize fields in China: meta-analysis
Source: Front Plant Sci. 2025 Feb 26;16:1493357. doi: 10.3389/fpls.2025.1493357 (PMC11897293; doi:10.3389/fpls.2025.1493357)

**Supporting Information**

**Impact of straw return on greenhouse gas emissions from maize fields in China-****meta analysis**

THREE FIGURES AND FOUR TABLES

List of contents

Tables:

Tab S1.Meta-analysis results indicate the effects of straw return on greenhouse gas emissions from cornfields............................................................................................................................................3

Tab S2 Results of subgroup analyses of CO_2_ emissions affected by straw return to fields...............4

Tab S3 Results of subgroup analyses of CH_4_ emissions affected by straw return to fields...............5

Tab S4 Results of subgroup analyses of N_2_O emissions affected by straw return to fields...............6

Figures:

Fig. S1 Funnel plot analysis of the effect of straw return on CO_2_ emission......................................7

Fig. S2 Funnel plot analysis of the effect of straw return on CH_4_ emission......................................8

Fig. S3 Funnel plot analysis of the effect of straw return on N_2_O emission......................................9

| Subgroup | est | low | hi | τ^2^ | Q | df | i^2^ |
| --- | --- | --- | --- | --- | --- | --- | --- |
| CO_2_ | 1.40 | 1.06 | 1.74 | 0.54 | 142.34 | 78 | 72.00% |
| CH_4_ | -0.04 | -0.47 | 0.40 | 2.25 | 219.76 | 84 | 61.80% |
| N_2_O | 0.40 | 0.06 | 0.75 | 1.75 | 282.69 | 116 | 59.00% |

Table S1 Meta-analysis results indicate the effects of straw return on greenhouse gas emissions from cornfields.

Note: est, effect value of GHGs; low, lower limit of 95% confidence interval; hi, upper limit of 95% confidence interval; τ^2^, degree of discrete heterogeneity of effect sizes; Q, observed variance; df, degrees of freedom; i^2^, proportionality of heterogeneity cases

| Subgroup | est | low | hi | τ^2^ | Q | df |
| --- | --- | --- | --- | --- | --- | --- |
| TN |  |  |  |  | 8.89 | 3 |
| <1 | 1.64 | 1.15 | 2.14 | 0.31 |  |  |
| 1-1.5 | 1.10 | 0.60 | 1.59 | 0.63 |  |  |
| >1.5 | 9.43 | 3.26 | 15.59 | 0.00 |  |  |
| SOC |  |  |  |  | 8.76 | 3 |
| <6 | 1.14 | 0.58 | 1.71 | 0.00 |  |  |
| 6-12 | 1.46 | 0.98 | 1.95 | 1.06 |  |  |
| >12 | 6.92 | 1.25 | 12.59 | 35.77 |  |  |
| Nitrogen rate |  |  |  |  | 4.78 | 3 |
| <150 | 2.66 | 1.33 | 4.00 | 2.52 |  |  |
| 150-250 | 1.24 | 0.80 | 1.68 | 0.66 |  |  |
| 250-350 | 1.60 | 1.00 | 2.21 | 0.00 |  |  |
| >350 | 4.52 | -4.17 | 13.20 | 46.18 |  |  |
| pH |  |  |  |  | 5.73 | 3 |
| <6 | 4.14 | 1.64 | 6.64 | 0.00 |  |  |
| 6-8 | 1.63 | 0.48 | 2.79 | 2.67 |  |  |
| >8 | 1.45 | 1.05 | 1.86 | 0.00 |  |  |
| Soil type |  |  |  |  | 16.15 | 6 |
| Cinnamon soil | 1.48 | 1.05 | 1.92 | 0.00 |  |  |
| Brown Soil | 0.94 | -0.13 | 2.00 | 2.14 |  |  |
| Saline-alkali soil |  |  |  |  |  |  |
| Fluvo-aquic soil | 1.85 | 0.99 | 2.71 | 0.88 |  |  |
| Black soil | 1.10 | 0.13 | 2.08 | 0.48 |  |  |
| Dark loessial soil | 2.38 | 1.01 | 3.74 | 0.00 |  |  |
| Red earth | 4.14 | 1.64 | 6.64 | 0.00 |  |  |
| Yellow brown earth | -2.72 | -5.66 | 0.22 | 0.00 |  |  |
| Purple soil |  |  |  |  |  |  |
| AAT |  |  |  |  | 6.09 | 3 |
| <10 | 2.36 | 1.25 | 3.47 | 2.37 |  |  |
| 10-15 | 1.12 | 0.74 | 1.50 | 0.17 |  |  |
| >15 | 2.82 | 0.52 | 5.13 | 6.60 |  |  |
| Rainfall |  |  |  |  | 8.55 | 3 |
| <400 | 2.12 | 0.21 | 4.03 | 3.06 |  |  |
| 400-800 | 1.25 | 0.91 | 1.58 | 0.26 |  |  |
| >800 | 2.31 | 0.42 | 4.19 | 4.13 |  |  |
| Plant method |  |  |  |  | 0.01 | 1 |
| Crop rotation | 1.41 | 1.02 | 1.80 | 0.60 |  |  |
| Continue crop | 1.45 | 0.67 | 2.23 | 0.54 |  |  |
| Tillage method |  |  |  |  | 3.17 | 1 |
| No tillage | 3.25 | 1.17 | 5.33 | 4.87 |  |  |
| Tillage | 1.34 | 0.99 | 1.69 | 0.48 |  |  |

Table S2 Results of subgroup analyses of CO_2_ emissions affected by straw return to fields.

Note: est, effect value of CO_2_ emissions; low, lower limit of 95% confidence interval; hi, upper limit of 95% confidence interval; τ^2^, degree of discrete heterogeneity of effect sizes; Q, observed variance; df, degrees of freedom.

Table S3 Results of subgroup analyses of CH_4_ emissions affected by straw return to fields.

| Subgroup | est | low | hi | τ^2^ | Q | df |
| --- | --- | --- | --- | --- | --- | --- |
| TN |  |  |  |  | 9.62 | 3 |
| <1 | 0.25 | -0.47 | 0.98 | 2.65 |  |  |
| 1-1.5 | 0.12 | -0.47 | 0.70 | 1.93 |  |  |
| >1.5 | -1.71 | -3.45 | 0.03 | 14.86 |  |  |
| SOC |  |  |  |  | 4.42 | 3 |
| <6 | 0.45 | -0.42 | 1.33 | 1.15 |  |  |
| 6-12 | -0.02 | -0.55 | 0.51 | 2.13 |  |  |
| >12 | -1.51 | -3.17 | 0.14 | 3.55 |  |  |
| Nitrogen rate |  |  |  |  | 0.65 | 3 |
| <150 | -0.06 | -0.79 | 0.66 | 1.63 |  |  |
| 150-250 | -0.13 | -0.80 | 0.55 | 5.58 |  |  |
| 250-350 | 0.41 | -1.38 | 2.19 | 7.64 |  |  |
| >350 | -0.38 | -1.33 | 0.56 | 0.75 |  |  |
| pH |  |  |  |  | 7.61 | 3 |
| <6 | -1.60 | -3.75 | 0.56 |  |  |  |
| 6-8 | 0.94 | -0.26 | 2.14 | 4.06 |  |  |
| >8 | 0.11 | -0.49 | 0.71 | 1.72 |  |  |
| Soil type |  |  |  |  | 19.52 | 6 |
| Cinnamon soil | 0.12 | -0.48 | 0.72 | 1.88 |  |  |
| Brown Soil | 0.13 | -1.15 | 1.41 | 3.62 |  |  |
| Saline-alkali soil | 0.06 | -2.28 | 2.40 | 4.64 |  |  |
| Fluvo-aquic soil | -0.93 | -1.83 | -0.03 | 1.23 |  |  |
| Black soil | -0.92 | -2.50 | 0.65 | 2.70 |  |  |
| Dark loessial soil | 2.85 | 1.32 | 4.39 | 0.00 |  |  |
| Red earth |  |  |  |  |  |  |
| Yellow brown earth |  |  |  |  |  |  |
| Purple soil | -0.63 | -2.15 | 0.90 | 0.33 |  |  |
| AAT |  |  |  |  | 8.22 | 3 |
| <10 | 0.04 | -1.42 | 1.50 | 7.45 |  |  |
| 10-15 | -0.04 | -0.45 | 0.37 | 0.90 |  |  |
| >15 | -2.02 | -3.53 | -0.51 | 1.41 |  |  |
| Rainfall |  |  |  |  | 0.57 | 2 |
| <400 | 0.05 | -1.09 | 1.19 | 1.83 |  |  |
| 400-800 | -0.03 | -0.53 | 0.47 | 2.64 |  |  |
| >800 | -0.63 | -2.15 | 0.90 | 0.33 |  |  |
| Plant method |  |  |  |  | 0.04 | 1 |
| Crop rotation | -0.05 | -0.56 | 0.47 | 2.65 |  |  |
| Continue crop | 0.02 | -0.82 | 0.86 | 1.51 |  |  |
| Tillage method |  |  |  |  | 1.25 | 1 |
| No tillage | 0.84 | -0.82 | 2.50 | 4.33 |  |  |
| Tillage | -0.14 | -0.58 | 0.30 | 1.97 |  |  |

Note: est, effect value of CH_4_ uptake; low, lower limit of 95% confidence interval; hi, upper limit of 95% confidence interval; τ^2^, degree of discrete heterogeneity of effect sizes; Q, observed variance; df, degrees of freedom.

| Subgroup | est | low | hi | τ^2^ | Q | df |
| --- | --- | --- | --- | --- | --- | --- |
| TN |  |  |  |  | 23.79 | 3 |
| <1 | 1.25 | 0.61 | 1.90 | 2.75 |  |  |
| 1-1.5 | 0.17 | -0.25 | 0.60 | 0.85 |  |  |
| >1.5 | -1.12 | -1.83 | -0.42 | 0.03 |  |  |
| SOC |  |  |  |  | 12.24 | 3 |
| <6 | -0.84 | -1.87 | 0.20 | 1.81 |  |  |
| 6-12 | 0.73 | 0.31 | 1.15 | 1.71 |  |  |
| >12 | 0.88 | -0.48 | 2.25 | 3.06 |  |  |
| Nitrogen rate |  |  |  |  | 2.94 | 3 |
| <150 | 0.23 | -0.56 | 1.02 | 2.47 |  |  |
| 150-250 | 0.60 | 0.06 | 1.14 | 2.33 |  |  |
| 250-350 | 0.59 | -0.22 | 1.41 | 1.43 |  |  |
| >350 | -0.20 | -1.03 | 0.62 | 0.71 |  |  |
| pH |  |  |  |  | 9.82 | 3 |
| <6 | 1.44 | -1.31 | 4.19 | 3.96 |  |  |
| 6-8 | 0.06 | -0.54 | 0.66 | 1.40 |  |  |
| >8 | -0.20 | -0.93 | 0.53 | 2.76 |  |  |
| Soil type |  |  |  |  | 48.36 | 8 |
| Cinnamon soil | 0.26 | -0.30 | 0.82 | 1.81 |  |  |
| Brown Soil | 1.22 | 0.36 | 2.08 | 1.73 |  |  |
| Saline-alkali soil | 1.81 | 0.98 | 2.63 | 0.00 |  |  |
| Fluvo-aquic soil | -0.41 | -1.16 | 0.35 | 1.42 |  |  |
| Black soil | -0.38 | -1.01 | 0.25 | <0.0001 |  |  |
| Dark loessial soil | 2.93 | 1.37 | 4.49 | 0.00 |  |  |
| Red earth | 2.73 | 0.57 | 4.88 | 0.00 |  |  |
| Yellow brown earth | -1.88 | -4.22 | 0.46 | 0.00 |  |  |
| Purple soil | -0.96 | -2.14 | 0.23 | <0.0001 |  |  |
| AAT |  |  |  |  | 18.58 | 3 |
| <10 | 0.88 | -0.15 | 1.91 | 4.06 |  |  |
| 10-15 | 0.65 | 0.30 | 1.00 | 0.74 |  |  |
| >15 | -1.23 | -2.19 | -0.27 | 1.23 |  |  |
| Rainfall |  |  |  |  | 1.44 | 3 |
| <400 | 0.08 | -0.77 | 0.93 | 0.64 |  |  |
| 400-800 | 0.51 | 0.12 | 0.90 | 1.86 |  |  |
| >800 | -0.26 | -2.17 | 1.65 | 3.66 |  |  |
| Plant method |  |  |  |  | 0.02 | 1 |
| Crop rotation | 0.40 | 0.02 | 0.78 | 1.65 |  |  |
| Continue crop | 0.46 | -0.46 | 1.39 | 2.83 |  |  |
| Tillage method |  |  |  |  | 2.81 | 1 |
| No tillage | 1.83 | 0.03 | 3.64 | 7.83 |  |  |
| Tillage | 0.27 | -0.06 | 0.59 | 1.19 |  |  |

Table S4 Results of subgroup analyses of N_2_O emissions affected by straw return to fields.

Note: est, effect value of N_2_O emissions; low, lower limit of 95% confidence interval; hi, upper limit of 95% confidence interval; τ^2^, degree of discrete heterogeneity of effect sizes; Q, observed variance; df, degrees of freedom.

Fig. S1 Funnel plot analysis of the effect of straw return on CO_2_ emission


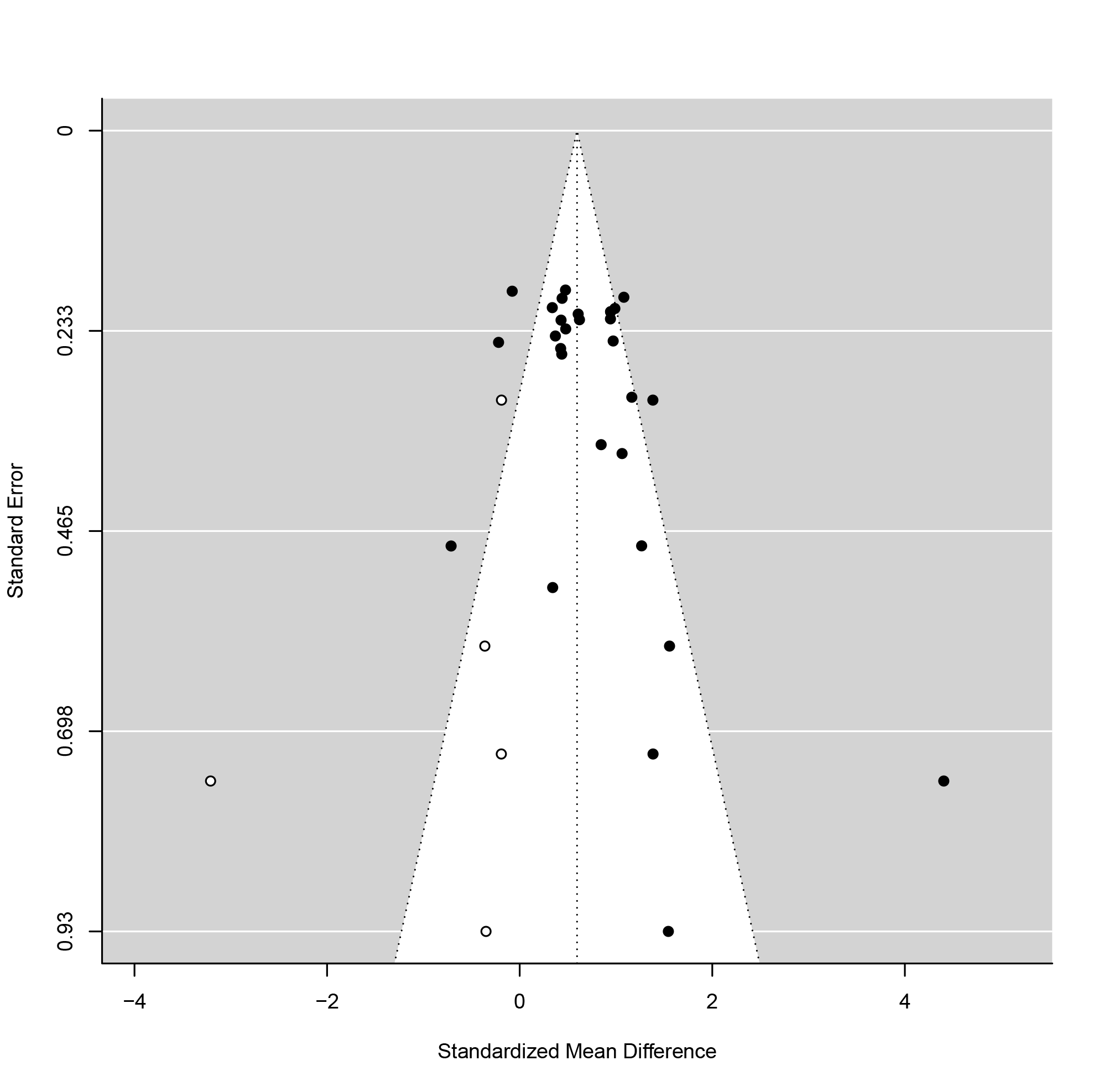


Fig. S2 Funnel plot analysis of the effect of straw return on CH_4_ emission


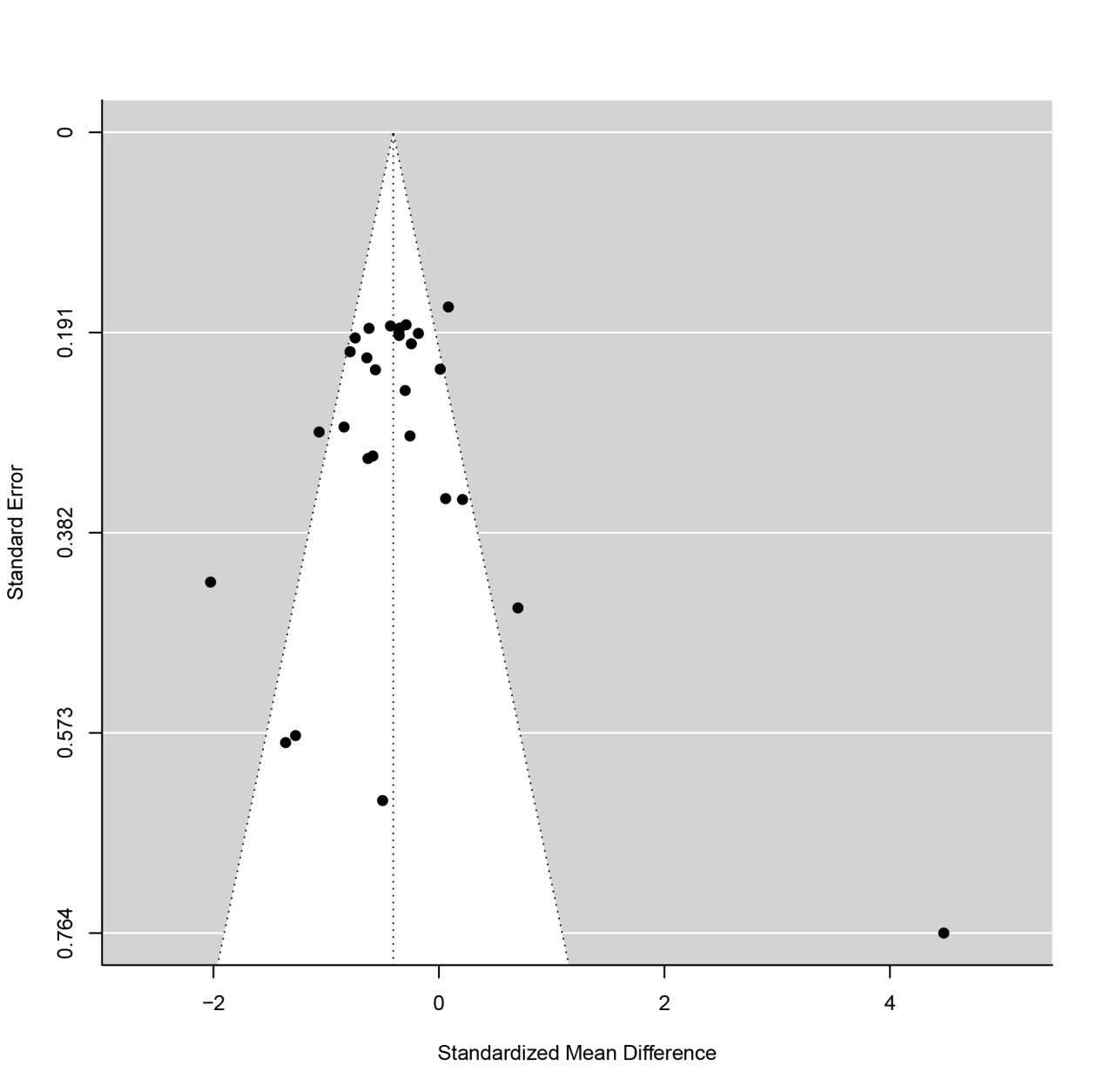


Fig. S3 Funnel plot analysis of the effect of straw return on N_2_O emission


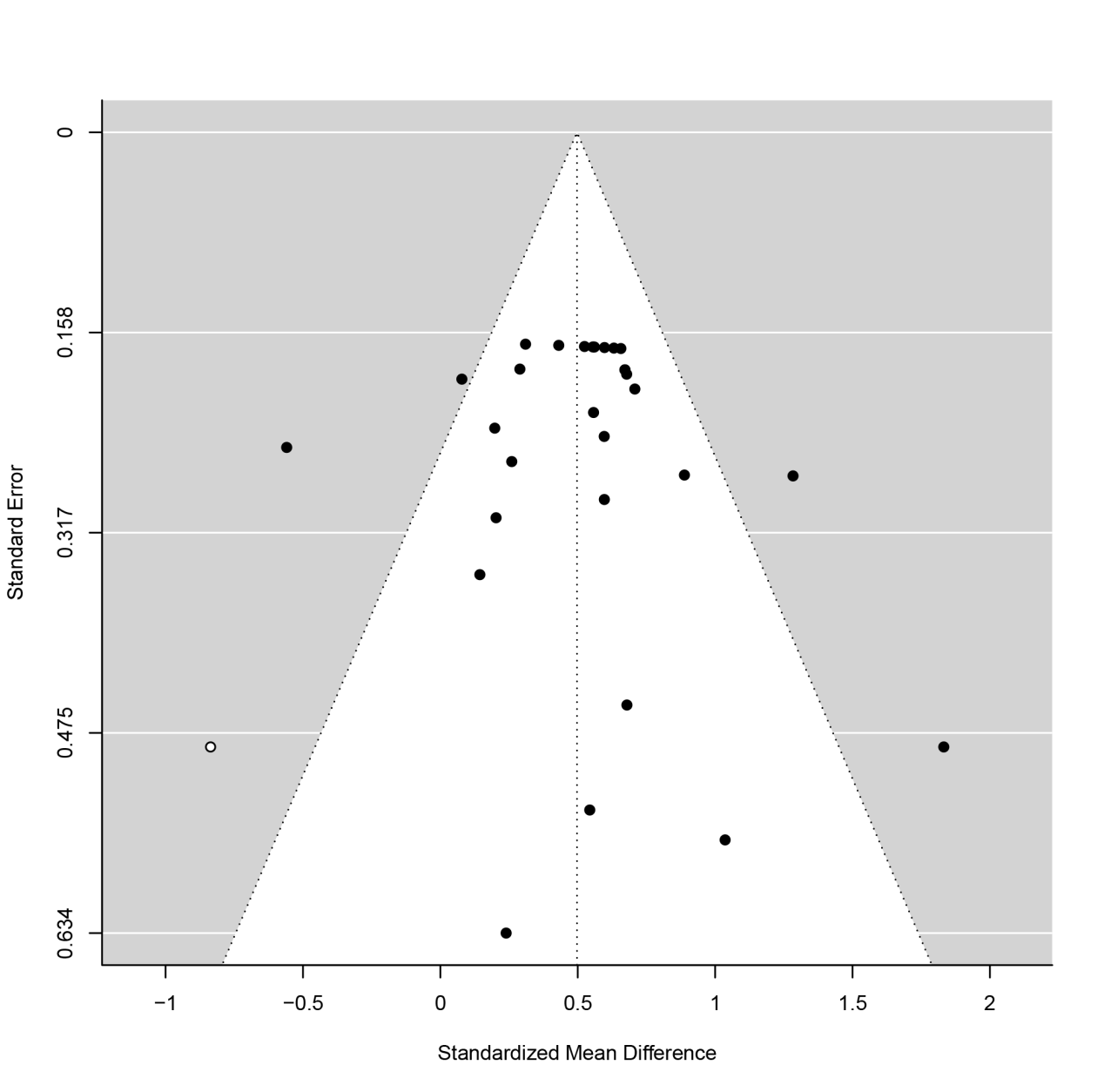

Supplement: Supplementary file 1 [file DataSheet1.docx]
